# Reprogramming tumor-associated macrophages in DMG/DIPG: emerging molecular and biophysical strategies

**DOI:** 10.3389/fimmu.2026.1788956

**Published:** 2026-03-17

**Authors:** Khatereh Khorsandi, Lynne El Ghorayeb, Elton VanNoy, Dalia Haydar

**Affiliations:** 1 Center for Cancer and Immunology Research, Children’s National Hospital, Washington, DC, United States; 2 The Institute for Biomedical Sciences, The George Washington University, Washington, DC, United States

**Keywords:** adoptive immunotherapy, diffuse intrinsic pontine glioma (DIPG), diffuse midline glioma (DMG), macrophage reprogramming, microglia, tumor microenvironment (TME), tumor-associated macrophages (TAMs)

## Abstract

Diffuse Midline Glioma (DMG), often formerly called Diffuse Intrinsic Pontine Glioma (DIPG) when in the brainstem, DMG/DIPG is a lethal pediatric brain tumor defined by infiltrative growth, resistance to conventional therapies, and a profound immunosuppressive tumor microenvironment (TME). Tumor-associated macrophages (TAMs), including resident microglia and infiltrating monocyte-derived macrophages, are the predominant immune population in DMG/DIPG. These cells adopt an immunosuppressive, pro-tumor state, promoting immune evasion and limiting the efficacy of therapies such as chimeric antigen receptor (CAR) T cells. Reprogramming TAMs toward a pro-inflammatory, anti-tumor phenotype offers a promising strategy to remodel the DMG/DIPG microenvironment. This review is the first to provide a comprehensive, integrative perspective on TAM-directed strategies in DMG/DIPG, spanning molecular, epigenetic, and biophysical approaches. We summarize TAM-mediated tumor progression and therapy resistance, and discuss molecular reprogramming strategies, including colony-stimulating factor 1 receptor (CSF1R) inhibition, microRNA-based circuits, and epigenetic modulators such as histone deacetylase (HDAC) and bromodomain and extra-terminal domain (BET) inhibitors. Nanoparticle-mediated delivery systems allow selective TAM targeting and enhanced blood-brain barrier (BBB) penetration. Additional strategies, including oncolytic viruses and macrophage-specific checkpoint blockade (e.g., CD47/SIRPα axis inhibitors), simultaneously promote tumor clearance and immune activation. We also highlight emerging biophysical approaches to modulate TAM function *in situ*. Photodynamic therapy (PDT) induces immunogenic cell death and pro-inflammatory macrophage activity, while focused ultrasound (FUS) transiently disrupts the BBB to enhance drug delivery and immune infiltration. Photobiomodulation and low-level light therapy (LLLT) may influence macrophage metabolism and phenotype, though their application in DMG/DIPG remains largely unexplored. Finally, we discuss combinatorial strategies integrating TAM reprogramming with CAR T cell therapy or chemotherapy to overcome the immunologically “cold” nature of DMG/DIPG. By uniting mechanistic insights with translational opportunities, this review establishes TAM reprogramming as a critical, underexplored frontier in DMG/DIPG immunotherapy, offering the potential to render an otherwise intractable tumor immunologically targetable.

## 1 Introduction

### 1.1 Overview of DMG/DIPG: incidence, prognosis, and treatment challenges

DMG/DIPG is an aggressive and fatal pediatric brain tumor that arises within the pons. Located in the brainstem, the pons is a critical region necessary for basic functions such as breathing, heart rate, and motor control (1). DMG/DIPG predominantly affects children between the ages of 5 and 10 years old, with an incidence of approximately 200–300 new cases annually in the United States (2). The prognosis for DMG/DIPG is dismal, with a median survival of less than one year from diagnosis, despite intervention and aggressive treatments. Currently, the standard of care consists of focal radiation therapy, which may temporarily alleviate symptoms but fails to significantly extend survival or eliminate the tumor (3). Surgical approaches are not viable due to the tumor’s location and infiltrative nature. The lack of targeted therapies, coupled with the highly invasive and heterogeneous nature of DMG/DIPG, poses significant challenges for developing effective treatments (4). DMG/DIPG’s resistance to therapy and rapid progression make it one of the most challenging cancers to treat, underscoring the need for novel therapeutic strategies (5).

### 1.2 The immunologically “cold” nature of DMG/DIPG

DMG/DIPG is often described as an immunologically “cold” tumor, a term referring to the absence of robust immune cell infiltration typically seen in other cancers. DMG/DIPG is characterized by a severe lack of cytotoxic T cell infiltration and a general absence of an adaptive immune response (6). The TME in DMG/DIPG is largely dominated by myeloid-derived cells, including TAMs and microglia, which adopt an immunosuppressive phenotype. These populations suppress T cell activation through inhibitory cytokine release and immune checkpoint signaling, leading to a lack of effective inflammatory responses against the tumor (7). Additionally, the failure of the adaptive immune system to recognize and respond to DMG/DIPG is partially due to the lack of sufficient tumor-antigen presentation by professional antigen-presenting cells (APCs) (8). As a result, DMG/DIPG remains largely resistant to immune checkpoint inhibitors and other immunotherapies that have shown promise in other cancers. This immunologically “cold” nature represents a significant barrier to the development of effective immune-based treatments for DMG/DIPG (6, 9).

### 1.3 TAMs within the TME

The TME of DMG/DIPG plays a crucial role in cancer progression and contributes to therapeutic resistance. The TME of DMG/DIPG is largely dominated by TAMs, which have been shown to exert a significant influence on tumor growth, immune evasion, and resistance to therapy (10). We acknowledge that macrophage and myeloid phenotypes are highly plastic and do not fit neatly into discrete categories; for clarity, we will generally use “M1-like” to refer to pro-inflammatory states and “M2-like” to refer to pro-tumorigenic states. TAMs in DMG/DIPG, which consist of both brain-resident microglia and infiltrating monocyte-derived macrophages, primarily exhibit an M2-like immunosuppressive phenotype. M2-like macrophages contribute to tumor progression by secreting growth factors and cytokines that promote angiogenesis, tissue remodeling, and immune suppression. Furthermore, TAMs in DMG/DIPG inhibit the activity of cytotoxic T cells and natural killer (NK) cells, thereby dampening the adaptive immune response and providing a shield for the tumor against immune-mediated destruction (11, 12). Understanding the role of TAMs in shaping the TME is critical for developing strategies to overcome the immune suppression inherent in DMG/DIPG and enhancing the efficacy of immunotherapy.

### 1.4 Rationale for targeting TAMs: bridging innate and adaptive immunity

Targeting TAMs in DMG/DIPG offers an exciting opportunity to bridge the gap between the innate and adaptive immune systems. While conventional immunotherapies such as CAR T cell therapy and checkpoint inhibitors focus on enhancing the activity of T cells, their efficacy in DMG/DIPG is hindered by the suppressive TME and the lack of immune cell infiltration. TAMs, as the dominant immune cell type in the DMG/DIPG microenvironment, can be reprogrammed to promote a pro-inflammatory, M1-like phenotype, which may help activate the adaptive immune response. By converting TAMs into cells capable of secreting pro-inflammatory cytokines, enhancing antigen presentation, and promoting T cell recruitment, it may be possible to overcome the immune suppression that characterizes DMG/DIPG and improve the effectiveness of existing immunotherapies. Additionally, targeting TAMs may offer the potential to disrupt other pro-tumorigenic pathways within the TME, including those involved in tumor angiogenesis, tissue remodeling, and resistance to chemotherapy. By reprogramming TAMs to shift from immunosuppressive to pro-inflammatory states, the innate immune system can effectively activate and recruit T cells, bridging innate and adaptive immunity. This is a promising strategy for transforming DMG/DIPG from an immune-excluded tumor into one that is responsive to immune-based therapies, offering hope for more effective treatments for this devastating disease (13).

## 2 The tumor microenvironment in DMG/DIPG

DMG/DIPG remains one of the most formidable challenges in pediatric oncology. Beyond its infiltrative nature and anatomical inaccessibility, DMG/DIPG is further protected by a uniquely immunosuppressive TME. A deeper understanding of the TME, particularly its myeloid cell composition, is essential for developing next-generation immunotherapeutic strategies. This section focuses on the cellular and functional immune architecture of the DMG/DIPG microenvironment, highlighting TAMs as key modulators of immune suppression and therapeutic resistance (14).

### 2.1 Immune landscape of DMG/DIPG

Unlike adult glioblastomas, which exhibit variable but occasionally substantial lymphocyte infiltration, DMG/DIPG tumors are consistently characterized by an immunologically “cold” microenvironment. Comprehensive analyses, including immunohistochemistry and single-cell RNA sequencing, have shown that T cells are notably and consistently scarce in DMG/DIPG tissue. Limited chemokine expression for T cell recruitment, insufficient co-stimulatory signaling, and an immunosuppressive cytokine milieu hinder the infiltration and activation of cytotoxic lymphocytes. In stark contrast, the immune compartment of DMG/DIPG is dominated by myeloid-lineage cells, particularly macrophages and microglia. Studies have shown that myeloid cells can constitute up to 30–50% of the total tumor mass in DMG/DIPG. These cells are not passive bystanders but active participants in supporting tumor growth, mediating immune evasion, and shaping the suppressive TME. The predominance of myeloid cells in DMG/DIPG also aligns with poor immunotherapy responses, as the absence of pre-existing T cell activity undermines the efficacy of strategies like immune checkpoint blockade (15, 16).

### 2.2 Characterization of TAMs in DMG/DIPG: microglia vs. monocyte-derived macrophages

**Figure 1:**
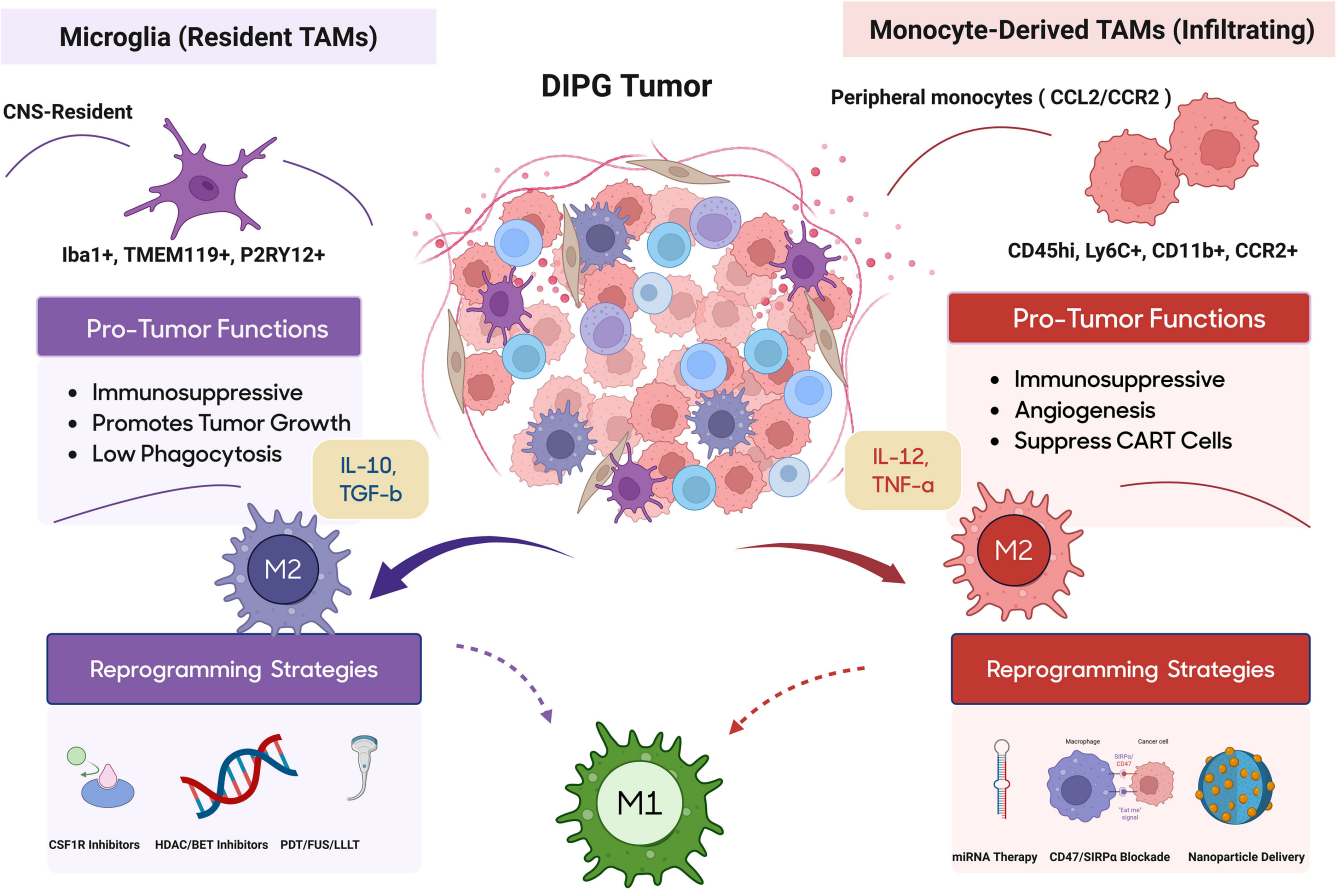
Distinct roles of resident microglia and monocyte-derived TAMsvin the DMG/DIPG tumor microenvironment. Microglia originate from the yolk sac and predominantly exhibit immunosuppressive, tumor-supportive functions, while monocyte-derived TAMs infiltrate from the circulation and contribute to angiogenesis, immune suppression, and therapy resistance. The figure highlights key markers, functional differences, and emerging reprogramming strategies aimed at shifting TAMs toward a pro-inflammatory, anti-tumor phenotype.

TAMs in DMG/DIPG comprise a heterogeneous population primarily derived from two sources: resident microglia and infiltrating monocyte-derived macrophages (MDMs). While both are myeloid cells, they differ in ontogeny, gene expression profiles, and functional roles within the tumor. Microglia, the brain’s innate immune sentinels, originate from yolk sac progenitors and are established in the central nervous system (CNS) during early embryonic development (17) (**Figure 1**). In DMG/DIPG, microglia are more abundant in peritumoral and early-stage regions and display a partially activated phenotype. While generally less immunosuppressive than MDMs, tumor-associated microglia contribute to immune evasion and tumor maintenance through the secretion of anti-inflammatory cytokines such as transforming growth factor-β (TGF-β), interleukin-10 (IL-10), and IL-6, as well as trophic and growth-supportive factors including insulin-like growth factor-1 (IGF-1), brain-derived neurotrophic factor (BDNF), vascular endothelial growth factor (VEGF), and colony-stimulating factor-1 (CSF-1). These factors collectively promote tumor cell survival, dampen local immune activation, and support tumor-associated angiogenesis (18).

In contrast, MDMs are recruited from the peripheral blood via chemokine gradients (e.g., CCL2/CCR2 axis) and infiltrate the tumor during progression (19). These cells exhibit a highly plastic and reactive phenotype, often acquiring more potent immunosuppressive functions than microglia. They express high levels of M2-like-associated markers and play key roles in suppressing T cell activation, remodeling the extracellular matrix, and promoting angiogenesis. Recent single-cell and spatial transcriptomic studies have revealed that microglia and MDMs occupy distinct anatomical and functional niches within the tumor. While microglia tend to localize tumor margins, MDMs accumulate in the tumor core, where they interact more directly with malignant cells and contribute to local immunosuppression (20, 21).

Although TAMs are frequently discussed as a single functional population, they arise from two distinct developmental origins: embryonically derived resident microglia and peripherally recruited MDMs. Accurately distinguishing these populations is critical for understanding their respective roles in tumor progression and therapeutic response, yet remains experimentally challenging, particularly in the central nervous system and pediatric brain tumors such as DMG/DIPG (22).

Lineage-tracing approaches provide the most definitive method for distinguishing microglia from MDMs. Genetic fate-mapping models, such as CX3CR1^CreER or Sall1^CreER systems, allow permanent labeling of yolk sac–derived microglia during embryogenesis, enabling their discrimination from later-infiltrating monocytes (17). Similarly, bone marrow chimeras and parabiosis experiments have been used to track the contribution of circulating monocytes to the tumor macrophage pool. While these approaches offer high specificity, they are largely restricted to preclinical mouse models and may introduce confounding factors, such as irradiation-induced blood–brain barrier disruption or altered immune cell recruitment. Importantly, such strategies are not feasible in human DMG/DIPG samples, limiting their translational applicability. Recent lineage-tracing and transcriptional analyses have highlighted distinct ontogeny-driven differences between resident microglia and infiltrating monocyte-derived macrophages. Notably, microglia specifically repress integrin α4 (Itga4/CD49D), while bone marrow–derived macrophages maintain its expression. This differential regulation allows CD49D to serve as a reliable marker for distinguishing microglia from BMDMs across primary and metastatic brain malignancies in both murine and human systems (23).

In contrast, marker-based strategies are more commonly employed in both experimental and clinical settings. Microglia have historically been identified by the expression of markers such as TMEM119, P2RY12, and Sall1, whereas MDMs are often characterized by higher levels of CD45, CCR2, CD14, and Ly6C (in mice). However, these markers are not absolute. Within the tumor microenvironment, microglia can downregulate canonical homeostatic markers, while infiltrating macrophages may adopt microglia-like transcriptional features in response to CNS cues. As a result, marker overlaps and phenotypic plasticity can blur distinctions between populations, particularly in advanced tumors or under therapeutic pressure (24). Recent advances in single-cell RNA sequencing (scRNA-seq) and spatial transcriptomics have provided higher-resolution insights into TAM heterogeneity. These approaches enable classification of microglia and MDMs based on developmental gene signatures rather than single markers and reveal distinct spatial niches and functional states within tumors. Nevertheless, transcriptional convergence between microglia and MDMs in the tumor microenvironment can still complicate interpretation, and technical factors such as dissociation bias or limited spatial resolution may influence results (25).

Collectively, no single experimental strategy is sufficient to unambiguously distinguish microglia from MDMs across all contexts. Lineage tracing offers definitive origin-based classification but is limited to animal models, while marker-based and transcriptional approaches provide broader applicability at the cost of reduced specificity. Consequently, many studies adopt the inclusive term “TAM” to describe both populations, particularly when functional overlap outweighs ontological distinctions. Integrating multiple complementary approaches will be essential for accurately defining TAM subsets and tailoring macrophage-targeted therapies in DMG/DIPG.

Increasing evidence indicates that resident microglia and MDMs occupy distinct spatial niches within gliomas, including DMG/DIPG, where microglia are frequently enriched at tumor margins and perivascular regions, while MDMs preferentially accumulate within hypoxic tumor cores. The mechanisms underlying this differential localization likely involve a combination of selective recruitment and local microenvironment-driven reprogramming (26). Tumor cells actively secrete chemokines and growth factors that regulate the recruitment of peripheral monocytes into specific tumor regions. Signaling axes such as CCL2–CCR2, CSF1–CSF1R, and CXCL12–CXCR4 promote the infiltration and retention of circulating monocytes, particularly within hypoxic and necrotic tumor zones. These gradients can spatially guide monocyte entry through disrupted vasculature and perivascular niches, resulting in preferential accumulation of MDMs in tumor cores where they interact closely with malignant cells. In contrast, resident microglia, already embedded within the central nervous system parenchyma, are more abundant in peritumoral and invasive margin regions, where they respond rapidly to early tumor-derived signals and tissue damage (26). Beyond selective recruitment, local microenvironmental cues play a critical role in shaping the phenotype and function of infiltrating myeloid cells. Hypoxia, nutrient gradients, extracellular matrix composition, and tumor-derived metabolites such as lactate and adenosine can reprogram both microglia and MDMs toward distinct activation states depending on their anatomical location. For example, hypoxic tumor cores enriched in HIF signaling and metabolic stress tend to promote highly immunosuppressive, pro-angiogenic macrophage phenotypes characterized by elevated VEGF, arginase-1, and PD-L1 expression. Conversely, cells at tumor margins may retain more inflammatory or antigen-presenting features, reflecting exposure to different cytokines and metabolic environments (27). Recent single-cell and spatial transcriptomic studies suggest that ontogeny alone does not rigidly determine TAM function; rather, developmental origin and local environmental programming act synergistically to shape myeloid cell identity. Infiltrating monocytes may gradually acquire microglia-like transcriptional features upon entering the CNS, while resident microglia can adopt macrophage-like immunosuppressive programs under sustained tumor influence. This phenotypic convergence complicates strict classification based solely on origin and underscores the importance of spatial context in defining TAM behavior (27).

The spatial organization of myeloid populations has important therapeutic implications. TAMs localized in hypoxic tumor cores may be more resistant to immunotherapy and contribute disproportionately to immune suppression, whereas those at tumor margins may represent more accessible targets for reprogramming strategies. Understanding whether tumors actively direct specific myeloid populations to defined regions or whether local conditions reprogram infiltrating cells after entry will be essential for designing therapies that effectively target TAM heterogeneity. Strategies combining inhibition of monocyte recruitment with local reprogramming of resident microglia may ultimately provide the most effective approach for reshaping the immunosuppressive microenvironment in DMG/DIPG (28).

### 2.3 Functional status of TAMs in DMG/DIPG

Functionally, the TAM population in DMG/DIPG is skewed toward an M2-like, immunosuppressive phenotype, which supports tumor progression and impedes antitumor immunity. These M2-like TAMs express high levels of anti-inflammatory cytokines (IL-10, TGF-β), enzymes such as arginase-1 (Arg1), and scavenger receptors like CD163 and CD206. Their metabolic programming favors oxidative phosphorylation and fatty acid oxidation, consistent with an M2-like metabolic profile that promotes tissue repair and tumor tolerance rather than immune activation (29). This polarization is driven by tumor-derived signals, including CSF-1, TGF-β, and IL-6, as well as hypoxic conditions within the tumor core. These cues not only sustain the M2-like phenotype but also actively inhibit antigen presentation, phagocytosis of tumor cells, and the production of pro-inflammatory mediators. As a result, TAMs in DMG/DIPG fail to initiate or amplify adaptive immune responses, contributing to the scarcity of activated CD8+ T cells within the tumor and undermining the effectiveness of immunotherapeutic interventions (30, 31). Moreover, M2-like TAMs act as critical barriers to CAR T cell therapy, which has shown promise in preclinical DMG/DIPG models. By secreting immunosuppressive factors and expressing checkpoint ligands such as PD-L1, TAMs can directly inhibit the proliferation and effector function of engineered immune cells (32).

## 3 The role of TAMs in tumor progression

TAMs are key architects of the TME and play a pivotal role in promoting tumor progression in various malignancies, including DMG/DIPG. In the immunologically “cold” context of DMG/DIPG, TAMs are not merely reactive cells but active participants that foster immune suppression, facilitate tumor growth, and contribute to therapeutic resistance. Understanding the multifaceted roles of TAMs is critical to uncovering new vulnerabilities in this lethal pediatric brain tumor.

### 3.1 TAMs as drivers of tumor growth, immune evasion, and angiogenesis

TAMs in DMG/DIPG support tumor growth through a range of mechanisms. These macrophages secrete a variety of growth factors, including epidermal growth factor (EGF), transforming growth factor-β (TGF-β), and platelet-derived growth factor (PDGF), which can directly promote the proliferation, invasion, and migration of DMG/DIPG cells. By remodeling the extracellular matrix (ECM) through the release of matrix metalloproteinases (MMPs), TAMs also facilitate tumor cell infiltration into adjacent healthy tissue, which is a hallmark of DMG/DIPG pathology (33). Beyond providing trophic support, TAMs actively shape the tumor microenvironment by promoting tissue remodeling, tumor cell survival, and resistance to therapy. From an immunological perspective, TAMs establish a highly suppressive microenvironment that limits effective anti-tumor immune responses. They secrete elevated levels of immunosuppressive cytokines, including interleukin-10 (IL-10), TGF-β, and interleukin-6 (IL-6), which inhibit dendritic cell maturation, impair antigen presentation, and suppress effector T cell activation (34). These cytokines also contribute to T cell exhaustion and reduced persistence, posing a significant barrier to emerging immunotherapies such as CAR T cell therapy. TGF-β, in particular, has been shown to inhibit cytotoxic T cell proliferation and function while promoting expression of inhibitory receptors such as PD-1 and TIM-3. IL-10 further dampens co-stimulatory signaling and promotes regulatory immune phenotypes, while IL-6 contributes to chronic inflammatory signaling that reinforces immune dysfunction within the tumor microenvironment. Collectively, these cytokine networks create a milieu that diminishes T cell cytotoxicity and limits the durability of adoptive cell therapies following tumor infiltration.

They also express immune checkpoint molecules such as PD-L1 and B7-H3, further dampening T cell-mediated cytotoxicity. This immunosuppressive signaling, coupled with the limited antigen-presenting capacity of M2-like TAMs, fosters a state of immune tolerance that allows tumor cells to escape immune surveillance (35, 36). Despite their predominantly tumor-promoting functions, TAMs retain the capacity to contribute to anti-tumor immunity through phagocytosis and antigen presentation under appropriate conditions. Reprogrammed macrophages can engulf tumor debris, process tumor-associated antigens, and present them via major histocompatibility complex molecules to T cells, thereby promoting epitope spreading and amplification of adaptive immune responses (37). However, in DMG/DIPG, these antigen-presenting functions are often suppressed by local cytokines and metabolic cues. Therapeutic strategies aimed at restoring macrophage phagocytic and antigen-presenting capacity—such as blockade of the CD47–SIRPα axis or activation of CD40 signaling—may enhance endogenous immune activation and improve the efficacy of CAR-T and other immune-based therapies (37). Given their central role in immune suppression, TAMs represent an attractive therapeutic target for combination immunotherapy. Approaches that skew TAMs toward pro-inflammatory, anti-tumor phenotypes include inhibition of colony-stimulating factor-1 receptor (CSF1R) signaling, targeting of PI3Kγ pathways, and activation of toll-like receptor (TLR) or CD40 signaling. These interventions can promote the production of pro-inflammatory cytokines such as IL-12, enhance antigen presentation, and support T cell activation (38). Alternatively, selective depletion of highly suppressive TAM subsets or blockade of inhibitory pathways such as PD-L1 and CD47 may further relieve immune suppression within the tumor microenvironment. Integrating TAM-targeted reprogramming or depletion strategies with CAR-T cell therapy holds considerable promise for overcoming immune resistance and enhancing therapeutic efficacy in DMG/DIPG.

Additionally, TAMs are potent mediators of angiogenesis, which is essential for sustaining tumor growth in the hypoxic environment of DMG/DIPG. They secrete VEGF and other pro-angiogenic factors that stimulate the formation of abnormal, leaky vasculature. This not only supplies nutrients and oxygen to the growing tumor but also creates niches of hypoxia that reinforce M2-like TAM polarization in a positive feedback loop (39). The resultant vascular irregularities can hinder drug delivery, further complicating treatment strategies. Collectively, TAMs shape the DMG/DIPG microenvironment into one that favors tumor progression through direct trophic support, ECM remodeling, suppression of adaptive immunity, and angiogenesis.

### 3.2 TAMs and resistance to therapy: barriers to immunotherapy and CAR T cells

**Figure 2:**
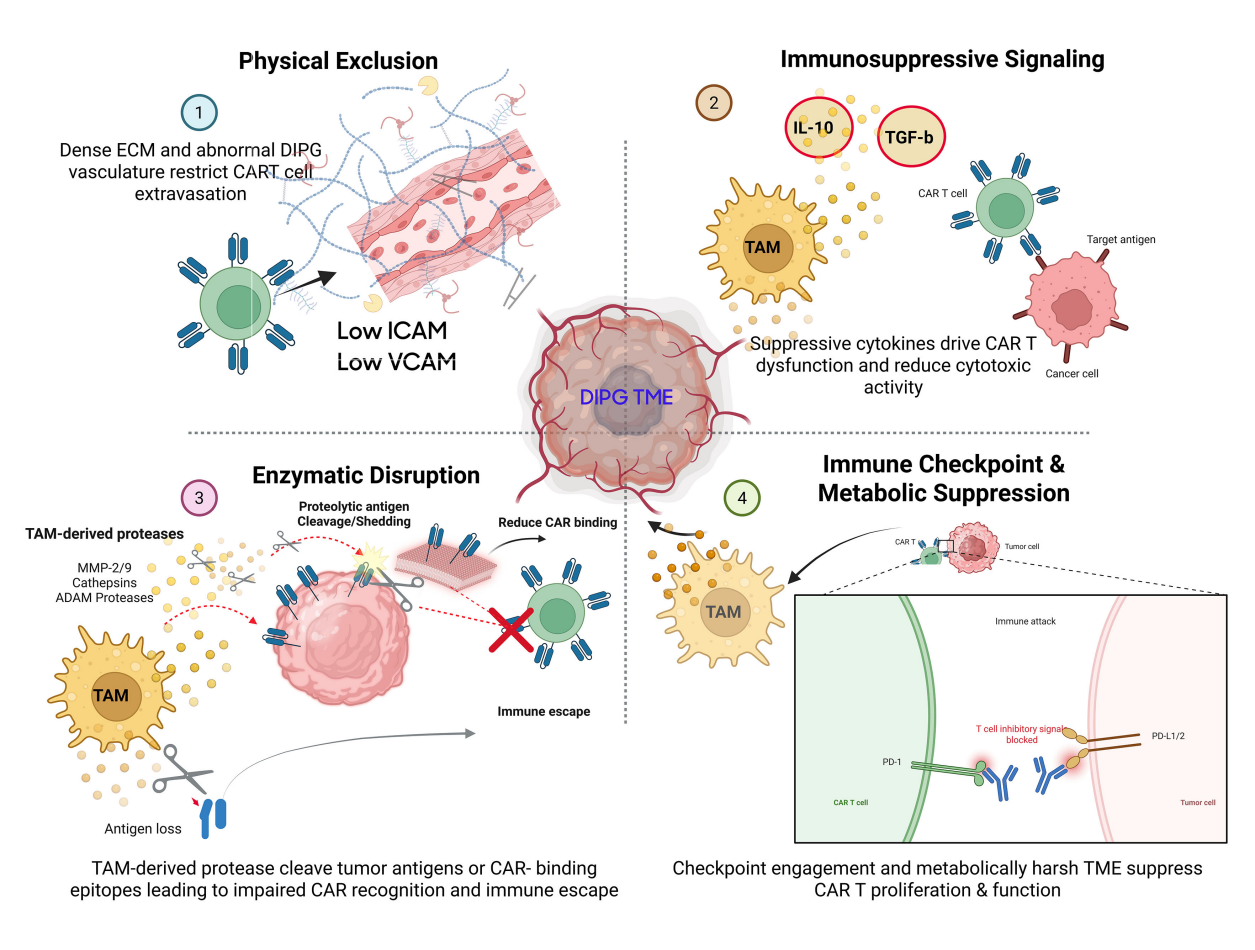
TAM-mediated barriers to CART therapy in DMG/DIPG. CAR T cell efficacy is limited by (1) physical exclusion due to tumor architecture (2), immunosuppressive signaling leading to exhaustion and apoptosis (3), enzymatic disruption of CAR–antigen engagement, and (4) immune checkpoint and metabolic suppression collectively driving therapeutic resistance in solid tumors.

The immunosuppressive functions of TAMs significantly impair the efficacy of emerging therapies, including immune checkpoint inhibitors and CAR T cell therapy, both of which rely on robust T cell activation and persistence within the TME. In DMG/DIPG, where T cells are already scarce, TAMs further compound the problem by inhibiting T cell infiltration, survival, and effector function (40). TAMs express PD-L1, Galectin-9, and CD47, molecules that interfere with T cell function or phagocytosis. Their secretion of arginase-1 and indoleamine 2,3-dioxygenase (IDO) depletes essential nutrients like arginine and tryptophan, further suppressing T cell metabolism and proliferation. These mechanisms blunt the effectiveness of checkpoint blockade, which has shown limited efficacy in DMG/DIPG clinical trials (16). In the context of CAR T cell therapy, which has shown preclinical promise against DMG/DIPG-specific antigens such as GD2 and B7-H3, TAMs present several barriers. First, they can physically obstruct CAR T cell infiltration by altering the tumor architecture. Second, TAM-derived TGF-β and IL-10 suppress CAR T cell cytotoxicity and proliferation. Third, TAMs can upregulate Fas ligand (FasL) and PD-L1, promoting CAR T cell exhaustion and apoptosis. Some studies also suggest that TAMs may secrete proteases that cleave surface CARs or their target antigens, thereby reducing CAR T cell engagement (41). Moreover, TAMs may play a role in promoting adaptive resistance. After initial tumor debulking by CAR T cells or radiation, TAMs may adopt a reactive, wound-healing-like phenotype that paradoxically supports tumor regrowth and repair. This contributes to a temporal evolution of the TME that ultimately limits the durability of therapeutic responses. In summary, TAMs not only facilitate DMG/DIPG progression through immunosuppressive and trophic mechanisms but also represent a major obstacle to effective treatment (42). Their inherent plasticity, however, makes them attractive therapeutic targets. Modulating TAM phenotype and function—either by skewing them toward a pro-inflammatory (M1-like) state or by selectively depleting immunosuppressive subsets—could significantly enhance the efficacy of existing and emerging therapies (**Figure 2**).

### 3.3 Additional modulators and emerging concepts of DMG/DIPG progression

#### 3.3.1 Role of other myeloid cells beyond TAMs

In addition to TAMs, other myeloid-derived populations contribute to immune regulation in gliomas, including dendritic cells (DCs) and myeloid-derived suppressor cells (MDSCs). DCs are essential for antigen processing, cross-presentation, and T cell priming; however, their abundance and functional maturity are markedly reduced in DMG/DIPG compared with adult gliomas. This paucity of functional DCs limits effective antigen presentation and contributes to weak adaptive immune activation. Conversely, MDSCs—both monocytic and granulocytic subsets—exert potent immunosuppressive effects through arginase-1 activity, nitric oxide production, and reactive oxygen species generation, thereby inhibiting T cell proliferation and effector function (43). Emerging evidence from pediatric glioma studies suggests that MDSCs may accumulate in response to tumor-derived cytokines and further reinforce immune evasion. Incorporating these myeloid subsets into future DMG/DIPG-focused studies may uncover additional immunoregulatory circuits and identify new targets for combinatorial immunomodulation (44).

#### 3.3.2 Tumor antigenicity and neoantigen landscape in DMG/DIPG

DMG/DIPG is characterized by a remarkably low tumor mutational burden and limited neoantigen diversity, contributing to its immunologically “cold” phenotype and resistance to immune-based therapies. The scarcity of tumor-specific antigens restricts effective T cell recognition and clonal expansion, thereby diminishing the efficacy of checkpoint inhibitors and endogenous anti-tumor immunity. Although recurrent mutations such as H3K27M provide shared targets, their low immunogenicity remains a challenge. Strategies aimed at enhancing tumor antigenicity—including synthetic neoantigen engineering, epigenetic modulation to increase antigen expression, or induction of epitope spreading through tumor cell death—represent promising avenues to overcome this barrier. A deeper understanding of the neoantigen landscape in DMG/DIPG will be critical for optimizing antigen-directed therapies, including CAR T cell approaches DMG/DIPG (45).

#### 3.3.3 Neural–immune crosstalk in the brainstem

DMG/DIPG arises within the brainstem, a region characterized by dense neuronal circuitry, high synaptic activity, and tightly regulated immune surveillance. This unique neuroanatomical context enables extensive neural–immune crosstalk that profoundly influences the tumor microenvironment and shapes anti-tumor immune responses. Neurons and glial cells actively communicate with immune cells through neurotransmitters, neuropeptides, and activity-dependent signaling pathways, many of which directly modulate the recruitment, polarization, and function of tumor-associated microglia and macrophages (46). Neurotransmitters such as glutamate and γ-aminobutyric acid (GABA), along with neuropeptides including substance P and neurotensin, have been shown to regulate myeloid cell behavior by engaging cognate receptors expressed on microglia and macrophages (47, 48). These signals can suppress pro-inflammatory activation, promote M2-like polarization, and enhance the secretion of immunosuppressive cytokines such as IL-10 and TGF-β (49). In the context of DMG/DIPG, where neuronal hyperexcitability and synaptic remodeling are increasingly recognized features, heightened neuronal activity may further bias TAMs toward tumor-supportive phenotypes. Emerging evidence from glioma models indicates that malignant cells can form functional synapse-like connections with neurons, allowing neuronal activity to directly promote tumor growth. This neuron-driven signaling also indirectly reinforces immune evasion by altering cytokine profiles, metabolic conditions, and immune checkpoint expression within the tumor microenvironment. Activity-dependent release of growth factors and metabolites may further stabilize immunosuppressive TAM states and limit effective T cell infiltration and persistence. The brainstem’s intrinsic requirement to tightly control inflammation—due to its role in vital autonomic and motor functions—adds layer of immune constraint. This region-specific immune privilege favors tissue-protective, anti-inflammatory responses, which tumors may exploit to evade immune-mediated clearance. Consequently, TAMs in DMG/DIPG are not only shaped by tumor-derived cues but also by ongoing neuronal signals that reinforce immune tolerance and suppress cytotoxic immunity (50). Understanding neural–immune crosstalk in DMG/DIPG has important therapeutic implications. Targeting neuro-immune signaling pathways, modulating neuronal activity, or disrupting neuron–tumor interactions may complement TAM-directed strategies and improve the efficacy of immunotherapies such as CAR T cells. Integrating neural biology into immunotherapeutic design may therefore be essential for overcoming the profound immune resistance characteristic of DMG/DIPG.

#### 3.3.4 Metabolic reprogramming of TAMs

Metabolic reprogramming has emerged as a central determinant of TAM function, influencing their polarization, immunoregulatory capacity, and interactions with tumor cells. TAMs display distinct metabolic phenotypes that closely correspond to their functional states. Classically activated, M1-like macrophages primarily rely on aerobic glycolysis and the pentose phosphate pathway to sustain rapid ATP production and support the production of pro-inflammatory mediators such as IL-12, TNF-α, and reactive oxygen species. In contrast, alternatively activated, M2-like macrophages—commonly associated with tumor promotion—preferentially utilize oxidative phosphorylation (OXPHOS), fatty acid oxidation (FAO), and lipid metabolism to maintain long-term survival and immunosuppressive activity (51). Within the tumor microenvironment, metabolic cues derived from tumor cells and stromal components further shape TAM polarization. Hypoxia, a hallmark of high-grade gliomas including DMG/DIPG, stabilizes hypoxia-inducible factors (HIF-1α and HIF-2α), which drive transcriptional programs that promote angiogenesis, immunosuppression, and metabolic adaptation. HIF signaling enhances expression of VEGF, arginase-1, and PD-L1 in TAMs, reinforcing their pro-tumorigenic phenotype and suppressing cytotoxic T cell activity (44, 45). Concurrently, tumor-derived metabolites such as lactate, adenosine, and kynurenine accumulate in the microenvironment and further skew macrophages toward M2-like states by inhibiting glycolysis-dependent inflammatory responses and promoting oxidative metabolism (52). Lipid metabolism also plays a pivotal role in TAM function. Uptake of fatty acids and cholesterol from the tumor milieu supports mitochondrial respiration and activates transcription factors such as PPAR-γ and STAT6, which drive anti-inflammatory gene expression and tissue-remodeling programs. Enhanced lipid droplet formation and fatty acid oxidation have been linked to sustained TAM-mediated immune suppression and resistance to therapy in multiple solid tumors, including gliomas (48, 49). Additionally, disruptions in glucose availability within the tumor microenvironment may force macrophages to adopt alternative metabolic pathways, reinforcing oxidative metabolism and limiting their ability to mount effective pro-inflammatory responses (53). Emerging evidence suggests that metabolic competition between tumor cells and immune cells further contributes to immune dysfunction. Tumor cells with high glycolytic rates can deplete local glucose and oxygen, restricting metabolic resources required for T cell and M1-like macrophage activation. This metabolic deprivation promotes a tolerogenic environment dominated by immunosuppressive TAMs and exhausted T cells. In DMG/DIPG, where hypoxia and nutrient gradients are pronounced, such metabolic constraints are likely to play a significant role in shaping the immune landscape and limiting responsiveness to immunotherapy. Therapeutically, targeting TAM metabolism represents a promising strategy to restore anti-tumor immunity. Pharmacologic inhibition of fatty acid oxidation, modulation of glycolytic flux, or disruption of HIF signaling can reprogram TAMs toward pro-inflammatory phenotypes and enhance antigen presentation. These metabolic interventions may act synergistically with established molecular approaches, such as CSF1R blockade, PI3Kγ inhibition, or epigenetic modulation, to overcome TAM-mediated immune suppression. Furthermore, integration with emerging biophysical and immunomodulatory strategies—including photodynamic therapy (PDT), focused ultrasound (FUS), or oncolytic viruses—may amplify metabolic stress within the tumor microenvironment and promote immune activation. Although much of the current knowledge derives from adult glioma and solid tumor studies, defining the metabolic dependencies and vulnerabilities of TAMs specifically within DMG/DIPG remains a critical research priority. Advanced approaches such as single-cell metabolomics, spatial transcriptomics, and *in vivo* metabolic tracing could provide deeper insights into TAM metabolic heterogeneity and identify actionable targets. A comprehensive understanding of TAM metabolic reprogramming will be essential for designing rational combination therapies that integrate metabolic modulation with immunotherapy to overcome the profound immune resistance characteristic of DMG/DIPG (54, 55).

#### 3.3.5 Oncolytic viruses and TAM interactions

Oncolytic viruses (OVs) exert dual anti-tumor effects by inducing direct tumor cell lysis and reshaping the immune microenvironment. OV infection can activate innate immune signaling pathways, enhance antigen release, and promote M1-like polarization of TAMs. In DMG/DIPG, OV therapy holds promise as a means of converting an immunologically “cold” tumor into an inflamed, immune-permissive environment. Strategic combinations of OVs with CAR T cells, immune checkpoint blockade, or TAM-targeting agents may amplify anti-tumor immunity and improve therapeutic durability. A deeper understanding of OV–TAM interactions in DMG/DIPG will be essential for optimizing treatment design and timing (56).

#### 3.3.6 Spatial immunology and single-cell insights

Recent advances in single-cell RNA sequencing and spatial transcriptomics have revealed pronounced heterogeneity among immune cells within gliomas, including TAMs. In DMG/DIPG, TAMs appear to occupy distinct spatial niches, with immunosuppressive populations enriched in tumor cores and more reactive or inflammatory subsets localized to tumor margins. These spatially defined immune states likely influence therapeutic response and resistance. Mapping immune architecture at single-cell resolution provides critical insights into cell–cell interactions and may guide spatially targeted therapeutic strategies, such as localized drug delivery or region-specific immune modulation. Integrating spatial immunology into DMG/DIPG research will refine our understanding of immune dynamics and support the rational development of precision immunotherapies for DMG/DIPG (57).

## 4 Molecular strategies to reprogram TAMs

Reprogramming TAMs from a pro-tumorigenic (M2-like) to an anti-tumorigenic (M1-like) phenotype represents a compelling approach to overcoming immunosuppression in DMG/DIPG. Given the critical role of TAMs in supporting DMG/DIPG progression through immune evasion, angiogenesis, and therapy resistance, multiple molecular strategies have emerged to modulate their recruitment, polarization, and immune checkpoint function. This section reviews key approaches currently under investigation for TAM reprogramming in the context of brain tumors, with a focus on DMG/DIPG.

### 4.1 Targeting TAM recruitment and survival

One of the earliest and predominant strategies against TAMs involves preventing their accumulation within the TME. TAMs in DMG/DIPG are derived both from resident microglia and bone marrow-derived monocytes, with the latter being actively recruited to the tumor site. Targeting chemotactic signals and survival factors can reduce the immunosuppressive burden of TAMs.

#### 4.1.1 CSF1/CSF1R inhibitors

CSF1 and its receptor CSF1R are critical for macrophage proliferation, differentiation, and survival. In many solid tumors, including high-grade gliomas, the CSF1-CSF1R axis facilitates the recruitment and maintenance of M2-like TAMs (58). Inhibiting CSF1R has been shown to reduce the number of TAMs and reprogram remaining cells toward an M1-like phenotype. Preclinical studies using CSF1R inhibitors such as PLX3397 (pexidartinib) or BLZ945 have demonstrated reduced tumor growth and enhanced sensitivity to immune checkpoint inhibitors (59).

In DMG/DIPG, where myeloid cells dominate the immune landscape, CSF1R expression is enriched in TAM populations. Targeting this axis could simultaneously decrease TAM density and restore immunostimulatory conditions favorable for T cell activity and CAR T cell therapy. However, because microglia are CSF1R-dependent, systemic CSF1R blockade in the brain risks significant depletion/functional alteration of resident microglia. Microglia serve homeostatic and neuroprotective roles; prolonged depletion or functional suppression can cause neurotoxicity or alter brain repair. This is particularly important in a vulnerable structure like the pons (DMG/DIPG location). Preclinical studies show substantial microglial depletion after CSF1R inhibitors and region-specific effects mediated by CSF1 vs IL-34 ligand distribution (60).

#### 4.1.2 Therapeutic targeting of the CCR2–CCL2 signaling axis

C-C chemokine receptor 2 (CCR2) is another key mediator of monocyte recruitment to the TME. Ligand binding, particularly by CCL2 (MCP-1), triggers the migration of CCR2+ monocytes from the periphery into the tumor. Inhibitors of CCR2 or neutralizing antibodies against CCL2 have been used in glioblastoma models to block this recruitment pathway, leading to reduced TAM infiltration and improved responses to radiotherapy and immunotherapy. Although the specific role of the CCR2 axis in DMG/DIPG has not been fully elucidated, transcriptomic analyses of murine and patient-derived DMG/DIPG samples have revealed elevated levels of CCL2. This suggests that CCR2 antagonism may serve as a viable strategy to curb the influx of immunosuppressive macrophages, thus tipping the balance toward an anti-tumor TME (61).

### 4.2 Modulation of TAM polarization

In addition to reducing TAM numbers, redirecting existing TAMs toward a pro-inflammatory phenotype is critical. Several innate immune pathways and small RNAs have been implicated in this process, offering targets to modulate macrophage plasticity.

#### 4.2.1 Use of toll-like receptor agonists

Toll-like receptors are innate immune sensors that can reprogram TAMs. Agonists of TLR3 (e.g., poly I:C), TLR4 (e.g., LPS), or TLR7/8 (e.g., imiquimod) activate downstream NF-κB signaling, promoting M1-like activation characterized by the production of IL-12 and TNF-α, as well as increased antigen presentation.

In preclinical models of brain tumors, intratumoral administration of TLR agonists has resulted in robust TAM reprogramming and synergistic effects with checkpoint blockade. Given the immunologically “cold” nature of DMG/DIPG, where T cell infiltration is limited, TLR-based therapies may serve as immune adjuvants, enhancing local inflammation and creating a permissive environment for T cell recruitment (62).

#### 4.2.2 Stimulator of interferon genes pathway activation

The STING pathway represents a potent avenue to induce type I interferon responses in TAMs. Upon recognition of cytosolic DNA, the cGAS-STING pathway activates interferon regulatory factors, promoting an M1-like phenotype and stimulating dendritic cell maturation and T cell priming (63). STING agonists such as ADU-S100 have shown encouraging results in preclinical glioma models, where they enhance macrophage-mediated phagocytosis and augment adaptive immune responses. While the efficacy of STING activation in DMG/DIPG remains under investigation, its potential to reshape the TME suggests promising applicability, particularly in combination with CAR T cells or radiation (64).

### 4.3 MicroRNAs-based approaches

miRNAs are endogenous, small (~22 nucleotides), non-coding RNAs that regulate gene expression by targeting mRNA transcripts for degradation or translational repression. In macrophages, miRNAs are central to determining activation status and functional phenotype, making them potent tools for reprogramming TAMs from an M2-like, immunosuppressive state to an M1-like, pro-inflammatory phenotype (65).

#### 4.3.1 Key miRNAs involved in TAM reprogramming

Several miRNAs have been identified as critical regulators of macrophage polarization. miR-155 is a master regulator of M1-like polarization. It enhances pro-inflammatory signaling by targeting suppressor of cytokine signaling 1 (SOCS1), a negative regulator of STAT1. Upregulation of miR-155 promotes the expression of M1-like markers such as TNF-α, IL-12, and inducible nitric oxide synthase (iNOS), facilitating anti-tumor activity (66).

miR-146a, while often characterized as anti-inflammatory, plays a context-dependent role. It acts to fine-tune TLR and NF-κB signaling pathways, and in certain tumor microenvironments, its modulation can reduce chronic immunosuppression and restore homeostasis in TAMs (67).

miR-124 has been shown to suppress M2-like-associated genes like STAT3 and promote antigen presentation, contributing to immune activation in glioma models. It may also synergize with immune checkpoint blockade by enhancing macrophage responsiveness (68).

miR-21 and miR-223 are frequently associated with M2-like phenotypes. Their inhibition has been proposed as a strategy to reduce TAM-mediated immune suppression and support reprogramming toward an M1-like state (69).

In the context of DMG/DIPG, studies examining the miRNA landscape of TAMs remain limited, but extrapolation from high-grade glioma (HGG) and glioblastoma (GBM) models suggests a significant opportunity for miRNA-based interventions, especially in combination with CAR T cells or STING agonists (70).

#### 4.3.2 Delivery systems for miRNA therapeutics

A major challenge in using miRNAs therapeutically lies in their delivery to target cells, especially in the CNS, where the BBB presents a formidable obstacle. Several innovative delivery platforms are being explored to overcome this limitation:

Nanoparticles (NPs): Cationic liposomes, polymer-based nanoparticles (e.g., PLGA), and gold nanoparticles have been engineered to encapsulate and protect miRNAs while facilitating their uptake by TAMs. These systems can be surface-functionalized with ligands or antibodies (e.g., CD206-binding peptides) to selectively target M2-like macrophages within the DMG/DIPG microenvironment (71).

Exosomes: These naturally occurring extracellular vesicles offer an immunologically inert, biocompatible delivery system. Engineered exosomes can be loaded with synthetic miRNAs or antagomirs (miRNA inhibitors) and directed toward TAMs. Glioma-derived or monocyte-derived exosomes have shown promise in preclinical models, especially when delivered via intratumoral injection or convection-enhanced delivery (CED) (72).

Viral Vectors: Lentiviral and adeno-associated viral vectors (AAVs) provide high-efficiency gene transfer and sustained expression of miRNAs. In the CNS setting, AAVs with macrophage-specific promoters (e.g., CD68, CD11b) could be used to ensure selective modulation of TAMs while minimizing off-target effects on neurons and glia (73).

Collectively, miRNA-based reprogramming strategies represent a highly modular and adaptable platform for modulating the immune landscape in DMG/DIPG and may complement other immunomodulatory approaches, such as checkpoint blockade or CAR T cell therapy.

### 4.4 Epigenetic modulation

Epigenetic regulation has emerged as a key mechanism governing both tumor cell plasticity and the functional polarization of TAMs, making it an attractive target for combinatorial therapeutic intervention in DMG/DIPG. Epigenetic modifications—including DNA methylation, histone acetylation, and chromatin remodeling—can simultaneously influence tumor cell proliferation, immune evasion, and macrophage activation states. In diffuse midline glioma, recurrent histone mutations such as H3K27M drive widespread epigenetic dysregulation that promotes tumor growth and suppresses immune recognition. These tumor-intrinsic epigenetic alterations can also indirectly shape the tumor microenvironment by modulating cytokine secretion, metabolic signaling, and immune checkpoint expression, thereby reinforcing TAM-mediated immune suppression (74). Several epigenetic regulators exert shared effects across tumor and immune compartments. Histone deacetylase (HDAC) inhibitors, for example, can enhance tumor antigen expression and increase susceptibility to immune-mediated killing while simultaneously promoting macrophage polarization toward pro-inflammatory phenotypes characterized by increased antigen presentation and IL-12 production. Similarly, inhibition of enhancer of zeste homolog 2 (EZH2), a key mediator of histone methylation, has been shown to reduce tumor cell proliferation and reverse suppressive transcriptional programs in myeloid cells, thereby enhancing anti-tumor immunity. Bromodomain and extraterminal domain (BET) inhibitors can further modulate transcriptional networks that regulate inflammatory signaling, cytokine production, and immune checkpoint expression in both tumor cells and TAMs (75, 76). Despite these shared mechanisms, important cell-type-specific epigenetic effects must also be considered. In tumor cells, epigenetic therapies may primarily influence proliferation, differentiation state, and antigenicity, whereas in macrophages they can alter polarization, phagocytic activity, and cytokine secretion profiles. For instance, modulation of histone acetylation in macrophages can shift transcriptional programs from M2-like immunosuppressive states toward M1-like inflammatory phenotypes, while in tumor cells, similar interventions may increase expression of neoantigens or stress ligands that enhance immune recognition. Understanding these distinct yet interconnected epigenetic responses will be essential for optimizing therapeutic timing, dosing, and combination strategies (77).

Targeting epigenetic regulators, therefore, offers a dual opportunity to reprogram both tumor cells and the surrounding immune microenvironment. Combining epigenetic therapies with TAM-modulating approaches, CAR-T cell therapy, or immune checkpoint blockade may enhance antigen presentation, relieve immune suppression, and improve therapeutic responsiveness in DMG/DIPG. Further investigation into the shared and cell-type-specific epigenetic mechanisms governing tumor–immune interactions will be critical for developing rational combination therapies capable of overcoming the profound immune resistance characteristic of this disease (78) (79).

#### 4.4.1 HDAC inhibitors

HDAC inhibitors (e.g., vorinostat, panobinostat) function by promoting histone acetylation, leading to an open chromatin state and increased transcription of pro-inflammatory genes. In TAMs, HDAC inhibition has been shown to suppress M2-like-associated genes (e.g., ARG1, CD206), induce the expression of IL-12 and TNF-α, and promote antigen presentation by upregulating MHC-II and costimulatory molecules (80).

In brain tumor models, HDAC inhibitors have also been found to sensitize tumor cells to radiation and immune-mediated killing. Their ability to cross the BBB and modulate both tumor cells and the immune microenvironment makes them attractive candidates for DMG/DIPG, where immunological “coldness” and treatment resistance prevail (81).

#### 4.4.2 DNMT inhibitors

DNMT inhibitors such as decitabine and azacitidine function by incorporating into DNA and trapping DNMT enzymes, leading to passive demethylation of hypermethylated promoters and reactivation of silenced genes. In DMG/DIPG—particularly the H3K27M-mutant subtype characterized by global DNA hypermethylation, disrupted PRC2 function, and aberrant chromatin states—DNMT inhibition can restore expression of tumor suppressor pathways (e.g., p16, p21, p53 targets), increase MHC-I and MHC-II expression, elevate interferon-stimulated genes, and diminish the stem-like, immune-evasive transcriptional program that enables DMG/DIPG cells to escape immune surveillance (82). This epigenetic reactivation enhances tumor immunogenicity and may sensitize DMG/DIPG to T-cell–based therapies and immune checkpoint blockade. In TAMs, however, DNMT inhibitors mediate a distinct but complementary effect: rather than reversing tumor suppressor silencing, they undo the epigenetic repression that maintains the M2-like, anti-inflammatory macrophage phenotype. Demethylation in myeloid cells reopens access to M1-associated transcription factors such as IRF5 and STAT1, enables robust production of pro-inflammatory cytokines (TNF-α, IL-12), increases antigen-presentation machinery and co-stimulatory molecules (CD80, CD86), and reduces expression of M2 markers like Arg1, CD163, and CD206 (83). This reprograms TAMs from an immune-suppressive, tumor-supportive state toward an M1-like, immunostimulatory phenotype capable of enhancing T-cell trafficking, activation, and persistence. Importantly, because DMG/DIPG contains a disproportionately myeloid-dominant TME with extensive microglial involvement, DNMT inhibition must be carefully calibrated: while it may convert suppressive TAMs into allies, excessive or prolonged exposure risks unintended activation of resident microglia or broad inflammatory effects in the brainstem (84, 85). Nevertheless, the combined epigenetic reprogramming of both DMG/DIPG tumor cells (increasing vulnerability and antigenicity) and TAMs (enhancing inflammatory support and reducing suppression) creates a uniquely synergistic environment for immunotherapies (86). This dual mechanism makes DNMT inhibitors attractive candidates for combination strategies with CAR T cell therapy, where improved antigen presentation, decreased myeloid suppression, and increased tumor immunogenicity may collectively enhance CAR T cell activity in an otherwise profoundly immunosuppressive DMG/DIPG microenvironment.

#### 4.4.3 BET inhibitors

Bromodomain-containing proteins (e.g., BRD4) read acetylated histone marks and promote transcription of oncogenes and immune-suppressive pathways. BET inhibitors (e.g., JQ1, I-BET762) interfere with this process and have been shown to inhibit transcription of IL-10 and other M2-like-supporting cytokines, induce IFN-responsive genes in TAMs, and suppress tumor-promoting inflammation and angiogenesis (87).

In gliomas, BET inhibitors reprogram TAMs and enhance the efficacy of PD-1 blockade and other immunotherapies (32). While their use in DMG/DIPG is still emerging, their ability to modulate both tumor cells and the TME suggests high translational potential.

### 4.5 Immune checkpoint targeting on TAMs

In addition to influencing T cells, TAMs themselves express and respond to immune checkpoint signals that regulate their phagocytic and immunosuppressive functions. Targeting these checkpoints may unmask anti-tumor properties of TAMs and improve immune surveillance in DMG/DIPG.

#### 4.5.1 Anti-CD47/SIRPα axis

The CD47-SIRPα axis serves as a signal that inhibits macrophage-mediated phagocytosis. Many tumors, including pediatric gliomas, overexpress CD47 to evade immune clearance. Blocking CD47 or its receptor Signal Regulatory Protein Alpha (SIRPα) enhances the phagocytic activity of TAMs and promotes antigen presentation (88).

Clinical trials of anti-CD47 therapies (e.g., magrolimab) are ongoing in solid tumors and hematologic malignancies (89). Preclinical work in glioma has shown that anti-CD47 treatment leads to tumor regression and increased M1-like macrophage infiltration (90). Applying this strategy in DMG/DIPG may help shift TAMs toward a more aggressive anti-tumor role while enhancing the efficacy of T cell–mediated therapies.

#### 4.5.2 Galectin-9/TIM-3 pathway modulation

Galectin-9, a β-galactoside-binding lectin, interacts with the immune checkpoint receptor TIM-3, which is expressed on T cells, dendritic cells, and macrophages. In TAMs, TIM-3 signaling has been associated with M2-like polarization and reduced inflammatory cytokine production. Inhibiting the Galectin-9/TIM-3 axis may enhance TAM responsiveness to pro-inflammatory cues and support anti-tumor immunity (91).

Recent data from DMG/DIPG models suggest increased expression of TIM-3 and Galectin-9 within the tumor microenvironment, correlating with immune evasion. The same study reports that Galectin-9 (LGALS9), one of the main ligands for TIM-3, is “very highly expressed” in DMG/DIPG patient samples (bulk/in-silico data) alongside TIM-3, whereas another ligand, CEACAM-1, was nearly undetectable (92). Targeting this pathway could simultaneously relieve T cell exhaustion and reprogram TAMs, creating a dual immunotherapeutic benefit.

## 5 Biophysical modulation of the tumor microenvironment

### 5.1 Photodynamic therapy

PDT represents a unique and underexplored modality in the modulation of the TME, particularly through its capacity to alter immune cell behavior and stimulate innate and adaptive anti-tumor responses (93). Traditionally employed as a local ablative technique for surface and hollow organ tumors, PDT’s mechanism of action offers profound immunomodulatory potential that can be harnessed to reprogram TAMs. In DMG/DIPG, where therapeutic innovation is urgently needed, PDT emerges as a promising biophysical intervention.

#### 5.1.1 Mechanism of action of PDT in immunomodulation

PDT requires three essential components: a photosensitizer (PS), a specific wavelength of light, and molecular oxygen. Upon light activation, the photosensitizer transitions into an excited state and transfers energy to surrounding oxygen molecules, generating reactive oxygen species (ROS), including singlet oxygen (^1O_2), hydroxyl radicals, and hydrogen peroxide. These ROS cause direct cytotoxicity to tumor cells via membrane and mitochondrial damage, triggering apoptosis, necrosis, and autophagy (94).

More importantly, PDT induces immunogenic cell death (ICD) characterized by the release of damage-associated molecular patterns (DAMPs) such as HMGB1, calreticulin, ATP, and heat shock proteins. These molecules act as endogenous danger signals, facilitating the recruitment and activation of antigen-presenting cells (APCs), including macrophages and dendritic cells (DCs). The result is a localized inflammatory response with systemic immunological consequences. This is an ideal setting for shifting the immunosuppressive DMG/DIPG microenvironment toward an immunostimulatory one (95).

#### 5.1.2 Impact of PDT on TAMs

TAMs are highly plastic and responsive to environmental cues, including those triggered by ROS and DAMPs. Multiple studies have shown that PDT can exert direct and indirect effects on TAM phenotype and function.

PDT-induced oxidative stress and DAMP release can induce phenotypic changes, including a shift from M2-like, immunosuppressive TAMs into M1-like, pro-inflammatory macrophages (96). M1-like TAMs exhibit elevated expressions of IL-12, TNF-α, and iNOS, enhancing their cytotoxic capacity and promoting T cell activation. Additionally, PDT increases the expression of MHC class II and costimulatory molecules (CD80/CD86) on macrophages, thereby enhancing their antigen-presenting capacity. This leads to more effective priming of CD4^+ and CD8^+ T cells within the TME (97). Following PDT, the inflammatory milieu then facilitates recruitment of cytotoxic T lymphocytes (CTLs), natural killer (NK) cells, and neutrophils into the tumor bed. TAMs in this setting further amplify the immune response by secreting chemokines such as CXCL9/10 and CCL5. These effects have been demonstrated in various preclinical models, including glioblastoma, head and neck cancers, and sarcomas. Given the immune desert phenotype in DMG/DIPG, characterized by minimal T cell infiltration and myeloid cell predominance, PDT holds particular promise in reshaping the TME through myeloid reprogramming (98, 99).

#### 5.1.3 PDT in DMG/DIPG: challenges and opportunities

One of the primary limitations of PDT in brain tumors, especially those located in deep and eloquent brain structures such as the pons, is light delivery. The BBB and dense parenchymal tissue hinder the penetration of external light sources, and traditional transcranial illumination is insufficient for targeting DMG/DIPG tumors (100). However, recent advances in neurosurgical technologies offer promising solutions. Fiber-optic probes and interstitial light delivery systems, including laser catheters and endoscopic tools, have enabled convection-enhanced delivery (CED) of both photosensitizers and light into deep-seated tumors. Additionally, MRI-guided stereotactic systems and robotic-assisted neurosurgery have further improved the precision and safety of intracranial PDT, allowing selective illumination of tumor cores with minimal damage to surrounding brain tissue (101). Development of near-infrared (NIR) photosensitizers with longer wavelengths (700–900 nm) has also extended light penetration depths, improving feasibility in deeper tumors like DMG/DIPG (102).

#### 5.1.4 Novel strategy: combining PDT with macrophage-targeting nanoparticles

An exciting frontier lies in combining PDT with macrophage-targeted delivery systems to synergistically reprogram TAMs in situ (103). Nanoparticles functionalized with TAM-binding ligands (e.g., mannose or anti-CD206 antibodies) can selectively deliver photosensitizers or immune-modulatory agents to immunosuppressive M2-like macrophages within the DMG/DIPG microenvironment (104).

This combinatorial method offers a precise and controlled approach. Local ROS generation via PDT can reprogram TAMs already loaded with immunostimulatory cargo, such as miR-155 or STING agonists, promoting a robust M1-like transition. Spatially confined light activation allows for localized TAM reprogramming without inducing systemic inflammation, which is a critical safety consideration in pediatric patients (105).

Reprogrammed TAMs further attract and activate effector T cells, enhance vascular normalization, and reduce glioma cell stemness, creating a more favorable environment for adjunctive therapies like CAR T cells (106). Preclinical validation of such approaches in H3K27M mutant glioma models is needed, but the conceptual framework provides a roadmap for engineering immunologically responsive DMG/DIPG niches using biophysical tools.

### 5.2 Focused ultrasound: a biophysical strategy to reprogram TAMs in DMG/DIPG

The TME of DMG/DIPG presents formidable barriers to therapeutic intervention. Chief among these is the BBB, a highly selective structure that restricts the entry of most systemic therapies into brain parenchyma. In addition, DMG/DIPG tumors are dominated by immunosuppressive TAMs and exhibit limited infiltration of cytotoxic T cells, posing another layer of resistance to immune-based therapies. FUS is emerging as a promising biophysical tool to modulate both the physical and immunological landscapes of DMG/DIPG (107).

#### 5.2.1 Mechanism of FUS

FUS is a noninvasive technique that uses acoustic energy to deliver mechanical force to a targeted region in the brain. When used in conjunction with intravenously administered microbubbles (gas-filled contrast agents), FUS causes the microbubbles to oscillate within the cerebral vasculature. This mechanical activity exerts pressure on endothelial tight junctions, leading to a reversible and localized opening of the BBB. The transient disruption allows for enhanced penetration of large-molecule drugs, nanoparticles, and immune modulators into the brain (108).

**Figure 3:**
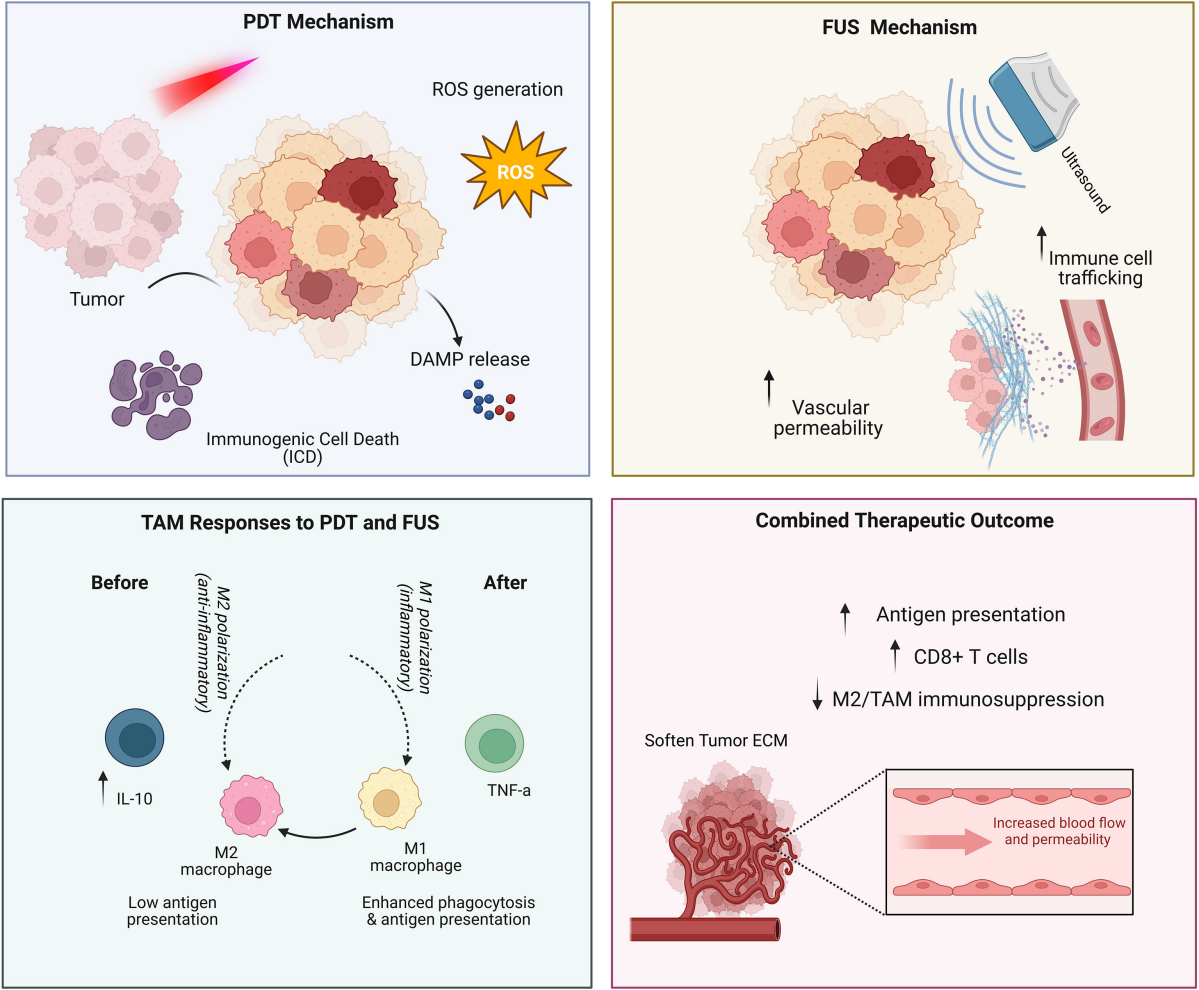
Biophysical modulation (PDT and FUS). PDT induces reactive oxygen species (ROS)–mediated immunogenic cell death and danger-associated molecular pattern (DAMP) release, while FUS enhances vascular permeability and immune cell trafficking. Together, these modalities reprogram tumor-associated macrophages toward an M1-like phenotype, increase antigen presentation and CD8^+^ T-cell infiltration, reduce M2-mediated immunosuppression, and improve therapeutic efficacy.

Beyond facilitating drug delivery, FUS also stimulates an immune response. The acoustic energy and resultant shear stress can lead to the release of danger signals and pro-inflammatory mediators within the tumor microenvironment, promoting a shift from immune tolerance to immune activation (**Figure 3**).

#### 5.2.2 Impact of FUS on TAM

Recent studies have shown that FUS can directly modulate TAM behavior, making it a powerful adjunct in strategies aimed at reprogramming the TME. FUS enhances the expression of inflammatory cytokines (e.g., TNF-α, IL-6) and chemokines (e.g., CCL2, CXCL10), which play roles in immune cell recruitment and activation. These molecules can attract circulating monocytes to the tumor site and influence their differentiation toward a pro-inflammatory phenotype (109). Additionally, FUS exposure has been reported to promote a shift in TAM polarization from the immunosuppressive M2-like phenotype to the pro-inflammatory M1-like state. M1-like TAMs produce nitric oxide and inflammatory cytokines that support anti-tumor immunity, improve antigen presentation, and enhance T cell activation. By disrupting the BBB and altering the chemokine landscape, FUS facilitates the infiltration of T cells, natural killer (NK) cells, and dendritic cells into the tumor. This increase in immune cell traffic synergizes with TAM reprogramming to potentiate an anti-tumor immune response (110).

#### 5.2.3 Preclinical successes: microbubble-assisted FUS in DMG/DIPG

Preclinical studies have demonstrated that microbubble-assisted FUS can transiently and safely open the BBB in the brainstem, enabling enhanced delivery of therapeutic agents to DMG/DIPG tumors that are otherwise inaccessible to systemic therapy. In patient-derived xenograft (PDX) models of H3K27M-mutant DMG/DIPG, MRI-guided FUS combined with microbubbles increased intratumoral accumulation of panobinostat approximately threefold, producing a 71% reduction in tumor volume and significantly prolonging survival (111). Similarly, in SU-DMG/DIPG-17 orthotopic xenografts, FUS enhanced the delivery of intravenously administered doxorubicin by roughly fourfold and suppressed tumor growth, demonstrating the utility of this approach for chemotherapeutics (112).

Beyond small molecules, FUS has been employed to improve the penetration of monoclonal antibodies and immune checkpoint inhibitors in glioma models. In preclinical glioma models (including non-DMG/DIPG brain tumors), FUS-mediated BBB opening increased PD-1 inhibitor delivery, enhanced CD4^+^ and CD8^+^ T cell infiltration, reduced tumor volume, and extended survival, suggesting potential applicability to DMG/DIPG immunotherapy (113). Additionally, the combination with nanoparticles further enhances intratumoral accumulation of immune-modulating agents, promoting a more inflamed and targetable tumor microenvironment (114).

Safety has been a consistent feature in these studies: repeated FUS + microbubble treatments in murine brainstem models did not induce hemorrhage, neuronal death, or motor deficits, supporting the translational potential of this modality in a delicate region such as the brainstem. Importantly, while preclinical successes are encouraging, outcomes vary depending on drug type, formulation, tumor model, and dosing schedule, emphasizing the need for systematic optimization, especially for biologics or nanoparticle-based therapeutics. Collectively, these advances underscore the promise of microbubble-assisted FUS as a strategy to overcome the anatomical and physiological barriers that have historically limited effective drug delivery to DMG/DIPG (115).

#### 5.2.4 Future direction: FUS combined with TAM-targeted immunotherapies

A particularly promising future direction is the combination of spatially precise, non−invasive blood–brain/blood–tumor barrier (BBB/BTB) opening by FUS with macrophage−targeted or immune−modulating therapies (116). In a murine glioma model, FUS-mediated BBB/BTB disruption significantly improved delivery and therapeutic efficacy of anti−CD47 antibodies, suppressing tumor growth and extending survival (117) (118). Additionally, biomimetic microbubble– or nanoparticle−based carriers have been successfully used in combination with FUS to deliver chemotherapeutics in orthotopic glioma, indicating that more complex payloads (e.g., TAM−modulating agents, STING or TLR agonists) could also be delivered. Translating this to DMG/DIPG, where both anatomical constraints (intact BBB, brainstem location) and a myeloid−dominated immunosuppressive microenvironment limit conventional therapy, could allow for efficient delivery of macrophage−targeted drugs (e.g., anti−CD47, CSF1R inhibitors) or immune−modulating nanoparticles, thereby reprogramming the TME, reducing TAM−mediated immunosuppression, and enhancing CAR−T or T cell–based therapies. However, because experimental evidence is largely derived from non−DMG/DIPG glioma models, and because DMG/DIPG has unique microenvironmental and anatomical features, rigorous preclinical studies in DMG/DIPG models are needed to assess delivery efficiency, dosing, safety, especially neuroinflammation and toxicity in the brainstem, and immune phenotypes post−treatment (**Table 1**) (134).

**Table 1:** Representative molecular and biophysical strategies for TAM reprogramming combined with immunotherapy and conventional treatments across preclinical and clinical cancer models.

Category	Reprogramming strategy	Combination therapy	Translational stage	Disease model	Publications/clinical trials
CAR T Cell Therapy	FRβ CAR T	Tumor-specific CAR T Cells (Mesothelin CAR)	Pre-Clinical	Ovarian Cancer	(119)
	F4/80 CAR T	Anti-PD1 and Anti-Ly6C	Pre-Clinical	Solid Lung Tumors	(120)
	EGFRvIII CAR T	Polyinosinic-polycytidylic acid (TLR3 agonist)	Pre-Clinical	Colon and Breast Cancer	(121)
	CD123 CAR T	NKG2DL CAR T	Pre-Clinical	AML	(122, 123)
	Sorafenib	GPC3 CAR T	Pre-Clinical	Hepatocellular Carcinoma	(124)
Immunotherapy	CSF-1R inhibitor	Durvalumab (anti-PD-L1)	Completed Phase I Trial	Pancreatic Cancer	NCT02777710
	Sotigalimab (CD40 agonist)	Nivolumab (Anti PD-1)	Completed Phase II Trial	Melanoma	NCT03123783
Chemotherapy	CD47/PD-L1-targeting nanoparticles	Temozolomide	Pre-Clinical	Glioblastoma	(122)
	BL-8040 (CXCR4 Antagonist)	Pembroizumab with 5-FU/LV and Onivyde	Completed Phase IIa Trial	Metastatic Pancreatic Adenocarcinoma	NCT02826486
	Mitazalimab (anti-CD40)	mFOLFIRINOX	Active Phase 1b/2 Trial	metastatic pancreatic ductal adenocarcinoma	NCT04888312
	CD47 Blockade	Temozolomide	Pre-Clinical	Glioblastoma	(125)
Epigenetic Modulation	CD47 Blockade	HDAC Inhibitor	Pre-Clinical	Breast and Colorectal Cancer	(126)
Radiation	SP-2-225 (HDAC6 Inhibitor)	Radiotherapy	Pre-Clinical	Melanoma	(127)
	CD47 Blockade	Fractionated RT	Pre-Clinical	DMG and GBM	(128)
	CD47 Blockade	Radiotherapy	Pre-Clinical	Small Cell Lung Cancer	(129)
	CSF-1R Inhibition	Radiotherapy	Pre-Clinical	Glioblastoma	(130, 131)
FUS	MRI Guided Focused Ultrasound	Microbubbles/PDL1 Blockade	Pre-Clinical	Breast Cancer	(132)
	MRI Guided Focused Ultrasound	Microbubbles	Pre-Clinical	Glioblastoma	(133)

## 6 Combination approaches

The complexity of the DMG/DIPG microenvironment necessitates a multipronged therapeutic approach. Immunosuppressive populations, CAR such as TAMs, impair antitumor immunity and hinder the efficacy of emerging treatments like CAR T cell therapy. Combining TAM reprogramming strategies with conventional or advanced modalities such as immunotherapy, radiation, or chemotherapy offers a promising avenue to overcome these barriers and achieve durable responses.

### 6.1 Synergy with CAR T cell therapy

While CAR T cells have shown some promise in treating pediatric brain tumors, their efficacy in DMG/DIPG is significantly limited by poor infiltration into the tumor site and the suppressive TME. TAMs play a central role in this immune exclusion, secreting anti-inflammatory cytokines and expressing checkpoint ligands that blunt T cell activation. Reprogramming TAMs toward a pro-inflammatory, M1-like phenotype can alter the chemokine milieu and enhance antigen presentation, thereby facilitating deeper CAR T cell penetration and persistence within the tumor (41).

For instance, preclinical models have demonstrated that combining CSF1R inhibitors or TLR agonists with CAR T therapy leads to improved intratumoral T cell accumulation and tumor regression. Similarly, leveraging miRNA-based approaches (e.g., miR-155 delivery) alongside CAR T cells may support a more immune-permissive microenvironment. These synergistic effects are especially crucial in DMG/DIPG, where T cell entry is intrinsically low and immune suppression is profound (135, 136).

### 6.2 Integration with radiation therapy

Radiation therapy (RT) remains the standard treatment for DMG/DIPG, providing transient clinical benefit primarily through tumor cytotoxicity. Beyond direct tumor cell killing, RT can enhance tumor immunogenicity by inducing immunogenic cell death, increasing antigen presentation, and promoting dendritic cell activation. However, RT also triggers compensatory recruitment of myeloid populations, particularly M2-like TAMs, which may dampen long-term antitumor immunity and contribute to therapy resistance (137).

To overcome this, preclinical studies have explored combinatorial strategies targeting TAMs or macrophage polarization alongside RT:

HDAC inhibitors + RT: In H3K27M-mutant DMG/DIPG PDX models, treatment with panobinostat or other HDAC inhibitors combined with fractionated RT enhanced tumor cell apoptosis while reprogramming TAMs toward an M1-like phenotype. This dual effect increased pro-inflammatory cytokine production (IL-12, TNF-α) and improved the activation of tumor-infiltrating T cells, resulting in prolonged survival compared with RT alone (138).

CD47 blockade + RT: In orthotopic glioma models, anti-CD47 antibodies combined with RT increased macrophage-mediated phagocytosis of irradiated tumor cells. RT enhanced exposure of “eat-me” signals (e.g., calreticulin) on tumor cells, synergizing with CD47 inhibition to reduce tumor burden and increase overall survival (139).

CSF1R inhibitors + RT: Preclinical studies in murine glioma models show that CSF1R inhibition following RT reduces RT-induced M2-like TAM infiltration, decreases immunosuppressive cytokines (IL-10, TGF-β), and promotes cytotoxic T cell activity within the tumor microenvironment (140).

FUS-assisted RT + immune modulators: Early preclinical work suggests that combining RT with BBB-opening strategies such as microbubble-assisted FUS can improve intratumoral delivery of TAM-modulating agents or checkpoint inhibitors in brainstem tumors, enhancing the efficacy of RT in immune “cold” tumors like DMG/DIPG (116). These studies collectively indicate that radiation’s efficacy can be augmented by targeting TAMs or reprogramming the immune microenvironment, shifting post-RT macrophage responses from immunosuppressive toward pro-inflammatory, thereby enhancing downstream adaptive immunity. Moving forward, such combinations in DMG/DIPG-specific models could maximize the limited window of RT benefit and lay the foundation for durable immunotherapeutic strategies.

### 6.3 Augmenting chemotherapy

Chemotherapy remains a cornerstone in cancer therapy, including in pediatric high-grade gliomas, but its efficacy is often limited by TAMs. M2-like TAMs promote chemoresistance through secretion of survival factors (e.g., IL-10, TGF-β), extracellular matrix remodeling, and scavenging or inactivation of chemotherapeutic drugs.

Preclinical strategies to enhance chemotherapy efficacy via TAM modulation include:

CSF1R Inhibition + Chemotherapy: In glioblastoma mouse models, the CSF1R inhibitor BLZ945 combined with temozolomide reduced M2-like TAM infiltration and promoted repolarization toward an M1-like phenotype. This combination improved chemotherapeutic response, enhanced tumor cell apoptosis, and prolonged survival compared with temozolomide alone.

CD47 Blockade + Chemotherapy: Anti-CD47 antibodies paired with doxorubicin or temozolomide in murine glioma models enhanced macrophage-mediated phagocytosis of tumor cells. Chemotherapy-induced tumor stress exposed “eat-me” signals such as calreticulin, synergizing with CD47 blockade to reduce tumor burden (122).

TLR/STING Agonists Encapsulated in Nanoparticles + Chemotherapy: Nanoparticle delivery of TLR agonists or STING agonists in combination with cytotoxic drugs in glioma models polarized TAMs toward an M1-like phenotype, increased intratumoral T cell activation, and improved chemotherapeutic outcomes.

Epigenetic Modulation + Chemotherapy: HDAC inhibitors or DNMT inhibitors in combination with chemotherapy can reprogram TAMs, reduce immunosuppressive cytokine production, and enhance tumor cell sensitivity to DNA-damaging agents in high-grade glioma models. These examples illustrate that combining chemotherapy with TAM-targeted strategies not only overcomes microenvironment-mediated drug resistance but also promotes a pro-inflammatory TME that enhances systemic anti-tumor immunity. Translating these approaches to DMG/DIPG could sensitize these highly resistant tumors to chemotherapy while concurrently promoting immune engagement.

## 7 Challenges and future directions

Reprogramming TAMs in DMG/DIPG represents a compelling therapeutic strategy. However, translating preclinical insights into effective clinical interventions remains a formidable challenge. Despite encouraging data from *in vitro* studies and animal models, several biological, technical, and logistical barriers must be addressed to realize the full therapeutic potential of TAM modulation in pediatric brain tumors.

### 7.1 Translational barriers from bench to bedside

One of the most significant hurdles in implementing TAM–targeted therapies is the persistent discrepancy between preclinical models and human disease. While numerous studies demonstrate effective TAM depletion or reprogramming in murine glioma models, these findings often fail to translate into clinical benefit. A major contributor to this gap lies in species-specific differences between murine and human immune systems. Key immune regulatory molecules, cytokine signaling networks, and receptor–ligand interactions can differ substantially between species, influencing macrophage activation, antigen presentation, and T cell responses. Differences in Fc receptor biology, cytokine responsiveness, and immune checkpoint regulation may alter TAM behavior and therapeutic outcomes, thereby limiting the predictive value of murine models for human immunotherapy responses.

Additional translational limitations stem from the widespread use of xenograft models, which require implantation of human tumor cells into immunocompromised mice. Although these models enable evaluation of tumor growth and targeted therapies, the absence of a functional immune system prevents accurate modeling of endogenous tumor–immune interactions. Consequently, the dynamic interplay among TAMs, T cells, and other immune populations—central to the success of immunotherapies such as CAR-T cells—cannot be fully assessed. Even in humanized mouse models, incomplete immune reconstitution and cross-species incompatibilities in cytokine signaling restrict faithful recapitulation of the human tumor immune microenvironment. Moreover, differences in macrophage ontogeny, immune complexity, and tumor architecture between mice and humans further contribute to the limited translational success of TAM-targeted approaches. These challenges are particularly pronounced in DMG/DIPG, where the tumor microenvironment is uniquely immunosuppressive and shaped by the specialized neuroimmune features of the brainstem, conditions that remain difficult to reproduce in current preclinical systems.

To address these limitations, there is growing recognition of the need for more physiologically relevant human-based experimental models. Patient-derived organoids and three-dimensional human brain tumor cultures can better preserve tumor heterogeneity, spatial organization, and microenvironmental signaling compared with traditional *in vitro* or xenograft systems. When integrated with autologous or engineered immune components, these platforms provide valuable opportunities to investigate TAM–tumor–T cell interactions in a context that more closely reflects human disease. Advances in organoid technology, microfluidic systems, and organ-on-chip platforms may further enhance modeling of immune infiltration, cytokine gradients, and therapeutic responses, thereby helping bridge the translational gap between preclinical findings and clinical applications.

In addition to biological discrepancies between models and patients, effective delivery of TAM-modulating therapeutics across the BBB remains a major technical challenge. Systemic administration of agents such as CSF1R inhibitors, microRNA-based therapies, or toll-like receptor agonists often results in limited brain penetration and potential systemic toxicity. Innovative strategies, including FUS, convection-enhanced delivery, and nanoparticle-based carriers, offer promising avenues to enhance central nervous system drug delivery and improve targeting of TAM populations. However, their safety, precision, and long-term feasibility, particularly in pediatric populations, require further clinical validation. Addressing these biological and technical barriers will be essential for successfully translating TAM-targeted strategies from bench to bedside and for improving therapeutic outcomes in DMG/DIPG.

### 7.2 Pediatric-specific considerations

Any therapeutic intervention in DMG/DIPG must account for the vulnerabilities unique to the pediatric population. Children have developing immune systems that differ in composition and responsiveness from adults, and interventions that modulate immune activity must be carefully tailored to avoid unintended consequences such as autoimmunity or impaired development. Furthermore, the pediatric brain is undergoing rapid growth and neurodevelopment, necessitating stringent safety profiles for all new therapeutic modalities.

Another major limitation is the inaccessibility of tumor tissue in DMG/DIPG due to its location in the brainstem. This makes real-time assessment of immune responses and TAM dynamics challenging, restricting the ability to adjust therapies based on biological feedback. Minimally invasive methods, such as liquid biopsies or advanced imaging techniques, need to be developed and validated to monitor immune changes non-invasively (1).

The field currently lacks immunocompetent and clinically relevant DMG/DIPG models that accurately mimic human disease. Many studies rely on immunodeficient mice implanted with human tumor cells, which fail to model the complex interplay between the immune system and the tumor microenvironment. To advance the development of TAM-targeted therapies, syngeneic mouse models with intact immune systems and genetically accurate representations of DMG/DIPG (e.g., H3K27M mutation) are urgently needed (141).

Such models would allow researchers to better understand how TAMs evolve within the DMG/DIPG milieu, respond to therapeutic intervention, and interact with other immune and stromal cells. They also provide a platform for testing combination strategies (e.g., CAR T cells plus CSF1R inhibitors) in an environment that reflects the immunological reality of pediatric brain tumors.

### 7.3 Biomarkers for monitoring TAM activity

The lack of reliable biomarkers to monitor TAM activity and polarization *in vivo* poses a significant barrier to clinical translation. Current imaging techniques, such as MRI and PET, are insufficient to assess the functional state of TAMs. Novel biomarkers, whether circulating miRNAs, soluble immune mediators, or macrophage-specific metabolic signatures, must be identified and validated to monitor therapeutic responses accurately (142).

Advanced imaging approaches, such as macrophage-targeted radiotracers or reporter gene imaging, may provide deeper insight into TAM dynamics in response to therapy. Furthermore, multi-omics profiling (e.g., transcriptomics, epigenomics) of patient-derived tissues and fluids can aid in identifying predictive markers of TAM responsiveness and treatment outcomes.

## 8 Conclusion

DMG/DIPG remains one of the most challenging pediatric brain tumors to treat, largely due to its location, aggressive nature, and the profoundly immunosuppressive TME. The TAMs predominantly exhibiting an M2-like, immunosuppressive phenotype constitute a major component of the DMG/DIPG TME and play critical roles in tumor progression, immune evasion, and resistance to therapy. Given their plasticity and central position within the tumor immune landscape, TAMs have emerged as compelling therapeutic targets. Reprogramming TAMs from a tumor-supportive to a tumoricidal phenotype offers a promising strategy to reshape the DMG/DIPG immune microenvironment and improve therapeutic efficacy.

Extensive preclinical research has demonstrated the potential of molecular strategies to disrupt TAM recruitment and survival, modulate their polarization, and reverse their immune checkpoint-mediated suppression. Biophysical modalities add another dimension to TAM reprogramming by stimulating macrophage activation and immune infiltration across the BBB. However, despite these encouraging advances, significant hurdles remain before TAM-targeting strategies can be broadly applied in clinical settings. The unique challenges of pediatric brain tumors require carefully designed, safe, and effective approaches. Improved models incorporating the hallmark H3K27M mutation and a functional immune system are urgently needed to evaluate TAM-targeted therapies and their combinations with immunotherapies like CAR T cells.

The future of DMG/DIPG therapy will likely rely on integrative, multimodal treatment regimens that combine TAM reprogramming with established therapies. For example, re-educating TAMs to an M1-like phenotype can enhance the efficacy of CAR T cell therapies by improving immune cell infiltration and overcoming local immune suppression. Similarly, combining TAM modulation with radiation or chemotherapy may sensitize tumor cells and amplify anti-tumor immune responses. These synergistic strategies can potentially overcome the limitations of monotherapies and provide durable clinical benefits.

In conclusion, reprogramming tumor-associated macrophages offers a novel and promising avenue to tackle the immunosuppressive microenvironment of DMG/DIPG. Molecular and biophysical strategies to modulate TAM recruitment, polarization, and function are rapidly advancing and show potential to improve immune surveillance and therapeutic outcomes. Realizing this potential requires a concerted effort to overcome pediatric-specific challenges, develop better translational models, and integrate biomarker-driven clinical approaches. By embracing an integrative framework that targets both tumor cells and their immune niche, the field moves closer to unlocking effective, immune‑based therapies for DMG/DIPG— a critical unmet need for children affected by this devastating disease.

## Glossary

^1O_2_: Singlet Oxygen
AAV: Adeno-Associated Virus
APCs: Antigen-Presenting Cells
ARG1: Arginase 1
ATP: Adenosine Triphosphate
BBB: Blood–Brain Barrier
BET: Bromodomain and Extra-Terminal (motif/domain)
CAR: Chimeric Antigen Receptor
CCL2: C–C Motif Chemokine Ligand 2 (Monocyte Chemoattractant Protein-1, MCP-1)
CCL5: C–C Motif Chemokine Ligand 5
CCR2: C–C Chemokine Receptor 2
CD4^+^: Cluster of Differentiation 4–Positive T Cells
CD8^+^: Cluster of Differentiation 8–Positive T Cells
CED: Convection-Enhanced Delivery
cGAS: Cyclic GMP–AMP Synthase
CNS: Central Nervous System
CSF1: Colony-Stimulating Factor 1
CSF1R: Colony-Stimulating Factor 1 Receptor
CTLs: Cytotoxic T Lymphocytes
CXCL9/10: C–X–C Motif Chemokine Ligands 9 and 10
DAMPs: Damage-Associated Molecular Patterns
DCs: Dendritic Cells
DMG/DIPG: Diffuse Intrinsic Pontine Glioma
DNMT: DNA Methyltransferase
ECM: Extracellular Matrix
EGF: Epidermal Growth Factor
FUS: Focused Ultrasound
GBM: Glioblastoma
HGG: High-Grade Glioma
HDAC: Histone Deacetylase
HIF: Hypoxia-Inducible Factor
HMGB1: High-Mobility Group Box 1
HSPs: Heat Shock Proteins
H_2_O_2_: Hydrogen Peroxide
ICD: Immunogenic Cell Death
IL: Interleukin
iNOS: Inducible Nitric Oxide Synthase
LLLT: Low-Level Light Therapy
MCP-1: Monocyte Chemoattractant Protein-1
MDSCs: Myeloid-Derived Suppressor Cells
MHC-II: Major Histocompatibility Complex Class II
miRNA/miR: MicroRNA
MMPs: Matrix Metalloproteinases
MRI: Magnetic Resonance Imaging
NK cells: Natural Killer Cells
NPs: Nanoparticles
O_2_: Molecular Oxygen
PD-1: Programmed Cell Death Protein 1
PD-L1: Programmed Death-Ligand 1
PDGF: Platelet-Derived Growth Factor
PDT: Photodynamic Therapy
PDX: Patient-Derived Xenograft
PLGA: Poly(lactic-co-glycolic acid)
PS: Photosensitizer
ROS: Reactive Oxygen Species
OH: Hydroxyl Radical
SIRPα: Signal Regulatory Protein Alpha
SOCS1: Suppressor of Cytokine Signaling 1
STAT: Signal Transducer and Activator of Transcription
STING: Stimulator of Interferon Genes
TAMs: Tumor-Associated Macrophages
TGF-β: Transforming Growth Factor Beta
TLR: Toll-Like Receptor
TME: Tumor Microenvironment
TNF-α: Tumor Necrosis Factor Alpha
